# Knowledge of symptoms and risk factors of breast cancer among women: a community based study in a low socio-economic area of Mumbai, India

**DOI:** 10.1186/s12905-020-00967-x

**Published:** 2020-05-18

**Authors:** Ranjan Kumar Prusty, Shahina Begum, Anushree Patil, D. D. Naik, Sharmila Pimple, Gauravi Mishra

**Affiliations:** 1 Department of Biostatistics, Indian Council of Medical Research-National Institute for Research in Reproductive Health (ICMR-NIRRH), Jehangir Merwanji Street, Parel, Mumbai, 400012 India; 2 Department of Clinical Research, ICMR-NIRRH, Jehangir Merwanji Street, Parel, Mumbai, 400012 India; 3 Department of Preventive Oncology, Centre for Cancer Epidemiology, Tata Memorial Centre, Homi Bhabha National Institute, Mumbai, 400012 India

**Keywords:** Breast cancer, Risk factors knowledge, Signs and symptoms, India

## Abstract

**Background:**

Breast cancer (BC) is leading cancer among women in India accounting for 27% of all cancers among women. Factors that make the policymakers and public health system worried are rising incidence of breast cancer in India and more importantly high death rates among breast cancer patients. One of the leading causes of high breast cancer deaths is lack of awareness and screening leading to the late presentation at an advanced stage. Therefore, the current research aimed to understand the knowledge of breast cancer symptoms and risk factors among women in a low socio-economic area of Mumbai.

**Methods:**

A cross-sectional study was conducted at Prabhadevi, Mumbai and primary data was collected from 480 women aged 18–55 years. Structured questionnaire was used to collect quantitative data pertaining to awareness, signs and symptoms of breast cancer. Bivariate and multivariate regression techniques were used for understanding of the socio-demographic differentials in breast cancer awareness among women.

**Results:**

The study found that around half (49%) of the women were aware of breast cancer. The women who were aware of breast cancer considered lump in breast (75%), change in shape and size of breast (57%), lump under armpit (56%), pain in one breast (56%) as the important and common symptoms. Less than one-fifth of the women who were aware of breast cancer reported early menstruation (5.6%), late menopause (10%), hormone therapy (13%), late pregnancy (15%) and obesity (19%) as the risk factors for breast cancer. The multivariate regression analysis showed women who had more than 10 years of schooling (Adjusted Odds Ratio: 3.93, CI: 2.57–6.02, *P* < 0.01) were about 4 times more likely to be aware of breast cancer than women who had less than 10 years of schooling.

**Conclusion:**

In conclusion, knowledge of danger signs and risk factors of breast cancer were low among women in the community. This may lead to late detection of breast cancer among women in the community. Therefore, the study calls for advocacy and larger intervention to enhance knowledge of breast cancer among women in the particular region with a special reference to women with low education.

## Background

Cancer incidence and mortality are growing at a vigorous pace across the globe and this transition is most striking among emerging economies. Globally, one-fourth (25%) i.e. 2.1 million cases of all female cancer diagnosed in 2018 were of breast cancer [1]. It is most commonly diagnosed cancer among females in more than 150 countries. Out of these 150 countries, breast cancer is the leading cause of mortality among all female cancers in 100 countries. The recent GLOBOCAN 2018 report shows age-standardised breast cancer incidence rate per 100 thousand females was very high in Australia (94.2), Western Europe (92.6) and Northern Europe (90.1) whereas it was lowest in South–Central Asia (25.9) region. However, the mortality rate in South Asian countries is more or less similar with greater mortality rate among most developing countries [1].

In India, the age-adjusted incidence rate of breast cancer was 25.8 per 100,000 women making it leading cancer among Indian females in 2012 [2]. Although the incidence rate was lower than many developed countries, it’s rapidly rising in Indian cities and the mortality rates were more than the United Kingdom (UK) (12.7 in UK vs 17.1 in India per 100 thousand women) which had a high incidence rate of 95 per 100 thousand females. According to National Cancer Registry Programme and GLOBOCAN 2018, there were 1,62,468 new cases of breast cancer and 87,090 deaths were reported for breast cancer in India [3]. In addition, there is a huge spatial variation across the nation with highest rates found in North-Eastern Indian states and major metropolitan cities like Mumbai, New Delhi, Kolkata and Chennai [4]. Detection of malignancy at advanced stages mainly leads to high death rates in India [5–7]. Lack of knowledge of signs and symptoms is considered as one of the major reasons contributing to the late detection backed by cumbersome referral pathways for diagnosis, lack of proper regional centres for treatment, incomplete treatment due to high out of pocket expenditures and several socio-economic, geographical, and cultural barriers associated with women’s health [5, 6, 8, 9]. The high death among women suffering from breast cancer is a concern for the national policymakers in addition to the increasing incidence rate.

There are multiple demographic, social and biomedical risk factors of breast cancer. Age of the women, early age at menarche, delayed first birth and menopause, nulliparity, short duration lactation, use of birth control pills, obesity, excess consumption of fats, hormone replacements and more importantly women having family history are considered as significant risk factors of breast cancer by various epidemiological and clinical studies [10–12]. One of the meta-analysis by Vishwakarma et al. [10] carried on 24 observational studies stated that highest odds ratio (OR) obtained for risk of breast cancer was among those who never had breastfeeding (pooled OR 3.69, 95% Confidence Interval = 1.70–8.01), never married women (pooled OR = 2.29, 95% CI = 1.65–3.17), and nulliparous women (pooled OR = 1.58, 95% CI = 1.21–2.06) [10]. One of the studies in South India found higher risk of breast cancer in urban area than rural areas [11]. This study also reported that the odds of breast cancer among urban women which increased with increase in proportion of overweight or obese (BMI-body mass Index > 25), size of the waist (> 85 cm) and size of hip (> 100 cm) among both pre-menopausal and post-menopausal women. Another study in rural Maharashtra found that most of the breast cancer cases were confined to women aged 40–49 years, home makers and upper economic strata group. Further, this study found breast cancer risk was 8 times higher among unmarried women, 3 times more among nulliparous women, 2 times more likely among post-menopausal women, 10 times more among those who had never breastfed, 1.5 times higher among women who were exposed to hormonal contraceptives and 4.5 time more likely among women with history of ovarian diseases than in comparison to married, non-nulliparous, premenopausal, women who ever breastfed, who have not been exposed to hormonal contraceptives, and women without any ovarian diseases respectively [12]. There are also studies which found difference in exposure to different type of environmental pollutants as a risk factor to breast cancer [13].

Several studies focused on different preventive and curative interventions which were carried both internationally and in India [14–19]. Although breast cancer prevention remains a baffling task due to involvement of multiple cell types at multiple stages, most intervention literature on breast cancer suggested that modifiable risk factors may be prevented through promotion of healthy diet, regular physical activities, regulating alcohol consumption and controlling weight which is likely to reduce the incidence of breast cancer in longer time period [20]. Further, literature also suggest that delay in detection leads to poor survival and early detection leads to better and economic treatment [21–23]. The delays were most among the older women and were mainly due to poor knowledge of symptoms and erroneous belief related to breast cancer and it’s treatment [22]. Therefore, the present paper tries to understand the knowledge of signs, symptoms and risk factors of breast cancer among women in the study area of Mumbai.

## Methods

The study was concentrated to lower socio-economic area catered by Prabhadevi maternity home and health post which comes under Municipal Corporation of Greater Mumbai (MCGM). Mumbai has a mixed health care system, inclusive of services provided by local bodies, the government of Maharashtra and public trusts and private service providers. The MCGM runs a network of primary, secondary and tertiary level facilities through medical college and hospitals, municipal general hospitals and speciality hospitals, maternity homes, dispensaries and health posts. The primary healthcare services are provided by health posts and dispensaries whereas maternity home provides specialized delivery care. The health posts were established to provide primary health services mainly in slum areas. The Prabhadevi maternity home and health post provides both primary healthcare services and maternal health care to lower socio-economic population in the Prabhadevi area of Mumbai.

The data used for the current study came from primary data collected for baseline survey of a breast cancer intervention study. The tertiary cancer specialized hospitals bear most of the burden of screening and treatment of breast cancer in India. The primary healthcare facilities in India is not well equipped with required human resources and training for cancer screening leading to late detection of cancer. So, this intervention was to test screening of breast cancer at primary care level for early detection of breast cancer cases with the available resources at present. The Prabhadevi facility was chosen for this study because it is both women centric and provides primary health care services. The cross-sectional baseline survey was conducted during November 2018 to March 2019.

The details of inclusion and exclusion criteria, sample size, sampling procedure, data collection and analysis are given below:

### Inclusion criteria

Women between 18 and 55 years of age were included in the study.

### Exclusion criteria

Women who were already diagnosed with breast cancer and under treatment, pregnant women and lactating women were excluded from the study.

### Sample size

About 80% of women aged 30–50 years were aware of breast cancer in Vikhroli, Mumbai [17]. However, our study focused on women 18–55 years of women. One of the study in similar settings at Delhi found around half (53%) of the women (aged 14–75 years) were aware of breast cancer [15]. Thereby considering 53% prevalence, 5% level of significance and 20% non-response rate, the required sample size was calculated as 478. Information was collected from 480 women participants.

### Sampling procedure

The complete area under Prabhadevi maternity home and health post was identified through the map available with Municipal Corporation of Greater Mumbai (MCGM). This health post is located at G-South ward of Mumbai. With the help of MCGM record, the low-income group housings based on criteria set by Maharashtra Housing and Area Development Authority (MHADA) were identified. Around 76 thousand low income group community population (according to MHADA, Government of Maharashtra) is catered by Prabhadevi Maternity Home under Municipal Corporation of Greater Mumbai. The whole area with around 19 thousand households was divided into 16 sections of around 1000–1400 households based on areas covered by 16 Community Health Volunteers at the health post. Mapping and house listing of the selected area/community was done to prepare a list of households having eligible women. Systematic random sampling was used to select the 480 eligible women from the list**.** Kish grid method was used to select women in case more than one woman was found eligible in the selected household [24].

### Data collection tools (baseline)

The tools were divided into two sections a) socio-economic background of the participants b) knowledge about breast cancer with questions related to awareness and practices (See supplementary file). The socio-economic background section focused on collecting individual level information like age, education, religion, caste, marital status of the participants. The second section was used to assess the women’s knowledge regarding breast cancer, sign and symptoms, risk factors, Breast Self-Examination (BSE), and Clinical Breast Examination (CBE) using a structured questionnaire. Women participants were asked whether they had ever heard of breast cancer. Those who have heard of breast cancers were further asked about knowledge of breast cancer signs and symptoms, risk factors and current practices using closed response questions. The questionnaire was prepared using existing literature and in consultation with the study team as well as experts constituting of oncologists, gynaecologist, public health, and social scientist. The tools were translated to both Hindi and Marathi languages for the convenience of participants. These questions were pilot tested with 20 participants (10 Hindi and 10 Marathi questionnaires each) at a similar socio-economic setting of Mumbai. The results from this pilot testing were used for modification of the words for easy comprehension of the participants. The content validity was ensured through expert consultation and pilot testing of the questionnaire. The field investigators were trained for 1 day and made familiar with the questions and ways of asking the questions. The data was collected through face to face interview with participants. Regular back-checks were conducted at the office to ensure data quality. The response rate was 96% for this baseline study.

### Statistical analysis

Univariate and bivariate analysis were performed using percentage and median to know the profile of study participants, proportion of women who were aware of symptoms, risk factors and screening methods and socio-economic differential in those symptoms and risk factors. Multivariate logistic regression was used to know the socio-demographic predictors of breast cancer awareness among women in the study area. The data were analysed using IBM SPSS 26.0 packages.

### Dependent variables

Women were asked ‘Have you ever heard of breast cancer?’. The response ‘Yes’ is coded as 1 and response “No” was coded as 0. This is used as a proxy variable for breast cancer awareness. Bivariate and multivariate binary logistic regression analysis was performed to see the differential and predictors of awareness of breast cancer. The other dependent variables used were specific symptoms, signs and risk factors of breast cancer to see differential socio-economic characteristics.

### Independent variables

Different socio-economic variables like age, religion, caste, working status, marital status, and years of schooling of women were used as independent variables in this study.

### Ethical permission

The Indian Council of Medical Research-National Institute for Research in Reproductive Health (ICMR-NIRRH) Ethics Committee for clinical studies, Mumbai has approved this study in compliance with the Helsinki declaration. Written consent from the participants was obtained during data collection. The confidentiality of the data was maintained during all the stages of research- data collection, data cleaning, and dissemination of research results.

## Results

### Profile of the study participants

The median age of the participants was 39 years and 98% of the women ever attended school. The median year of schooling was 12 years. The religious composition showed 93% of women were Hindu, 3 % of women were Buddhist/Neo-Buddhist and the remaining 4 % were from Christian, Jain, Muslim religions. More than two-thirds of the women (69%) were from upper caste or no caste groups whereas one-fourth of them were Other Backward Classes (OBC) and around 6% of the women were Scheduled Caste or Scheduled Tribe (SCs/STs). Only 16% of the women were employed. Majority of women (84%) were married and 77% of them had at least one child.

### Breast cancer awareness

**Fig. 1:**
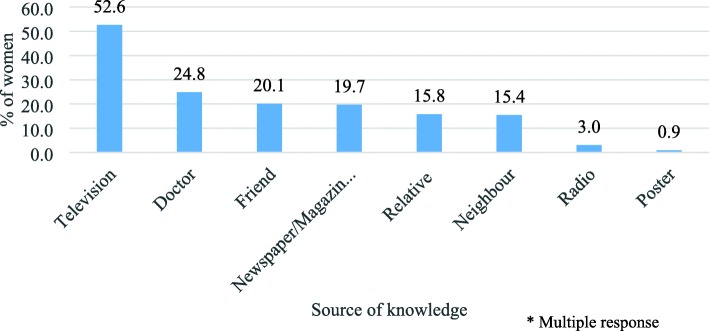
Different sources of knowledge of breast cancer among women (%) who were aware of it (N = 234)

About half (49%) of these women were ever heard of breast cancer. Breast cancer awareness was poor among women educated upto high school (10th) or not educated with only one-third of (34%) them ever heard of it. Nearly two-thirds of the women (61%) educated above 10th standard (higher education) were aware of breast cancer. Breast cancer awareness was better among middle aged women (25–34 years) than in comparison to younger (18–24 years) and older women (Table 1). Majority of these women had heard about breast cancer through television (53%) or from a doctor (25%) (Fig. 1).

**Table 1:** Differential in awareness of breast cancer among women 18–55 years by selected socio-demographic characteristics

Characteristics	Percentage	Adjusted Odds Ratio- With 95% C.I.	N
**Age Group (Years)**			
18–24	43.1	1	58
25–34	53.2	1.66 (0.72–3.83)	109
35–44	46.3	1.60 (0.65–3.94)	149
45–55	50.0	2.30 (0.94–5.67)	164
**Schooling**			
10 years or less	33.5	1	215
More than 10 years	66.1	3.93 (2.57–6.02)	265
**Religion**			
Hindu	48.5	1	445
Non-Hindu	51.4	1.19 (0.56–2.51)	35
**Caste**			
SC/ST/OBC	51.1	1	151
Others	47.7	1.24 (0.82–1.88)	329
**Family type**			
Nuclear	50.5	1	384
Joint/extended	41.7	0.68 (0.41–1.10)	96
**Employment**			
Not working	47.0	1	404
Working	57.9	1.57 (0.90–2.75)	76
**Marital status**			
Unmarried	45.5	1	77
Married	49.4	1.63 (0.75–3.51)	403
**Total**	**48.8**		**480**

a) N is Sample Size b) *SC* Scheduled Caste, *ST* Scheduled Tribe, *OBC* Other Backward Classes

### Multivariate analysis

The binary logistic regression analysis showed that education was the only significant predictor of breast cancer awareness (Table 1). The education of women was significantly and positively associated with awareness of breast cancer. The women who had more than 10 years of schooling (AOR: 3.93, CI: 2.57–6.02, *P* < 0.01) were about 4 times more likely aware of breast cancer than in comparison to women who had less than 10 years of schooling or no education.

### Knowledge of different signs and symptoms

**Fig. 2:**
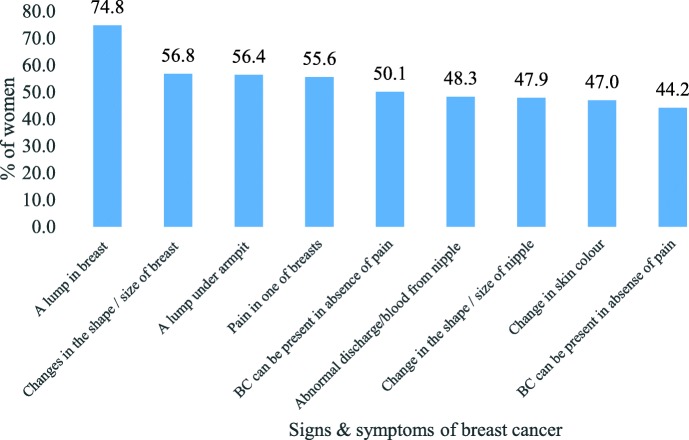
Percentage of women who had knowledge of different signs or symptoms of breast cancer

The knowledge of different symptoms among women ever heard of breast cancer (*N* = 234) is depicted in Fig. 2. Lump in breast was considered as a symptom of breast cancer by three-fourths of women. Interestingly, less than half of the women said abnormal discharge or blood from nipple (48%), change in shape or size of nipple (48%) and change in skin colour (47%) as symptoms of breast cancer. Only two out of five women (40%) thought breast cancer can be hereditary (not shown in figure).

The Table 2 shows the socio-economic differential in knowledge of danger signs of breast cancer among the women who were aware of breast cancer. The knowledge of different symptoms was less among marginalized classes like Scheduled Caste, Tribe and Other Backward Classes (SC or ST or OBC) group than in comparison to the other higher caste groups. A greater proportion of women, who were working had knowledge of different signs and symptoms of breast cancer than in comparison to women who were not working. It was also observed from the study that unmarried women had greater knowledge of all symptoms than in comparison to married women. No clear differential was found among age groups of women. Around half of the women believed ‘breast cancer means losing one’s breast’. Most women knew that breast cancer is not communicable (Table 3).

**Table 2:** Knowledge of danger signs of breast cancer among the women who are aware of breast cancer (*N* = 234)

Characteristics	Knowledge of Danger Signs of Breast Cancer										N
	Change in the shape/size of nipple	Pain in one of breasts	Abnormal discharge/blood from nipple	A lump in breast	Change in skin colour	A lump under armpit	Changes in the shape/size of breast	BC can be hereditary	BC can be present in absence of pain	BC is curable if detected in early stages	
**Age Group (Years)**											
18–24	60.0	64.0	56.0	76.0	52.0	64.0	64.0	44.0	60.0	68.0	25
25–34	46.6	60.3	51.7	74.1	50.0	51.7	60.3	41.4	53.4	75.9	58
35–44	46.4	49.3	49.3	73.9	40.6	55.1	50.7	40.6	49.3	63.8	69
45–55	46.3	54.9	42.7	75.6	48.8	58.5	57.3	37.8	51.2	58.5	82
**Schooling**											
10 years or less	31.9	43.1	35.8	65.3	38.9	44.4	41.7	30.6	47.2	55.6	72
More than 10 years	54.9	61.1	53.1	79.0	50.6	61.7	63.6	44.4	54.3	69.8	162
**Religion**											
Hindu	49.1	56.5	49.5	75.0	47.7	57.9	56.5	39.8	50.9	64.8	216
Non-Hindu	33.3	44.4	33.3	72.2	38.9	38.9	61.1	44.4	66.7	72.2	18
**Caste**											
SC/ST/OBC	45.5	53.2	46.8	70.1	39.0	44.2	49.4	39.0	48.1	66.2	77
Others	49.0	56.7	49.0	77.1	51.0	62.4	60.5	40.8	54.1	65.0	157
**Family type**											
Nuclear	50.5	55.2	50.0	73.7	50.5	59.3	57.2	43.3	51.5	61.9	194
Joint/extended	35.0	57.5	40.0	80.0	30.0	42.5	55.0	25.0	55.0	82.5	40
**Employment**											
Not working	45.8	52.1	46.3	71.6	45.3	53.2	51.6	37.9	47.4	62.1	190
Working	56.8	70.5	56.8	88.6	54.5	70.5	79.5	50.0	72.7	79.5	44
**Marital status**											
Unmarried	62.9	65.7	57.1	82.9	54.3	60.0	68.6	51.4	60.0	77.1	35
Married	45.2	53.8	46.7	73.4	45.7	55.8	54.8	38.2	50.8	63.3	199

*SC* Scheduled Caste, *ST* Scheduled Tribe, *OBC* Other Backward Classes

**Table 3:** Misconceptions related to danger signs of breast cancer among the women who are aware of breast cancer (*N* = 234)

Characteristics	Incorrect Knowledge of Danger Signs of Breast Cancer					N
	Woman with big breast get breast cancer	Use of antiperspirants or deodorants causes breast cancer	Trauma to breasts cause breast cancer	Breast cancer is communicable	Breast cancer means losing one’s breast(s)	
**Age Group**						
18–24	4.0	4.0	20.0	4.0	44.0	25
25–34	8.6	0.0	8.6	3.4	56.9	58
35–44	11.6	1.4	4.3	2.9	50.7	69
45–55	13.4	2.4	11.0	4.9	51.2	82
**Schooling**						
10 years or less	6.9	0.0	9.7	4.2	50.0	72
More than 10 years	12.3	2.5	9.3	3.7	52.5	162
**Religion**						
Hindu	10.2	1.9	10.2	4.2	52.8	216
Non-Hindu	16.7	0.0	0.0	0.0	38.9	18
**Caste**						
SC/ST/OBC	13.0	1.3	10.4	3.9	53.2	77
Others	9.6	1.9	8.9	3.8	51.0	157
**Family type**						
Nuclear	9.3	2.1	9.8	4.6	46.9	194
Joint/extended	17.5	0.0	7.5	0.0	75.0	40
**Employment**						
Not working	8.9	1.6	7.9	3.2	48.9	190
Working	18.2	2.3	15.9	6.8	63.6	44
**Marital status**						
Unmarried	2.9	2.9	17.1	5.7	42.9	35
Married	12.1	1.5	8.0	3.5	53.3	199

a) N is Sample Size b) *SC* Scheduled Caste, *ST* Scheduled Tribe, *OBC* Other Backward Classes

### Knowledge of risk factors

**Fig. 3:**
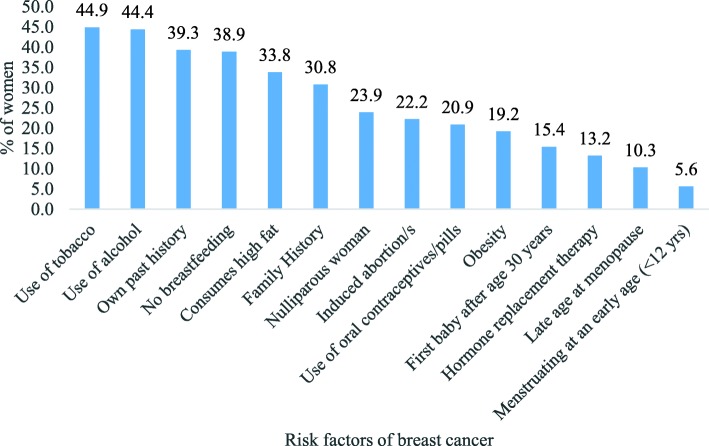
Percentage women who identified the risk factors of breast cancer

Understanding the risk factors of BC may help women in taking preventive measures. In this study, women who were aware of breast cancer (*N* = 234) were asked about the risk factors of breast cancer. The percentage of women who identified breast cancer risk factors are shown in Fig. 3. Most women believed consumption of excess tobacco (45%) and alcohol (44%) leads to breast cancer followed by risk factors like past history of BC (39%), no breastfeeding (39%), consumption of high fat foods (34%) and family history (31%). The knowledge of important biological risk factors like early age of menstruation (6%) and late menopause (10%) were very low among the women, although they had heard of breast cancer.

The socio-economic differentials showed that with an increase in age of women, the knowledge of different risk factors goes down (Table 4). Further, the risk factors knowledge was slightly higher among higher educated women compared to the women who had education till secondary school (10th standard). Women from nuclear family, not working and married woman had lower knowledge of most of the risk factors than in comparison to women from joint family, working and unmarried women respectively. However, the overall knowledge of risk factors was low among all women even though they are aware of breast cancer.

**Table 4:** Knowledge of risk factors among the women who are aware of breast cancer (N = 234)

	Started menstruating at an early age (< 12 yrs)	Woman having late age at menopause	Nulliparous woman	First baby after age 30 years	Woman used oral contraceptives/pills	Woman who is obese	Woman consumes high fat	Woman undergone hormone replacement therapy	Not given breastfeeding to the child	Family history of BC	Woman having past history of BC
**Age Group (Yrs)**											
18–24	16.0	12.0	28.0	20.0	28.0	32.0	32.0	28.0	32.0	32.0	44.0
25–34	5.2	13.8	25.9	22.4	20.7	22.4	41.4	12.1	50.0	32.8	44.8
35–44	2.9	8.7	20.3	10.1	20.3	11.6	30.4	14.5	36.2	29.0	34.8
45–55	4.9	8.5	24.4	13.4	19.5	19.5	31.7	8.5	35.4	30.5	37.8
**Schooling**											
10 years or less	2.8	8.3	18.1	9.7	22.2	15.3	36.1	9.7	37.5	20.8	40.3
More than 10 years	6.8	11.1	26.5	17.9	20.4	21.0	32.7	14.8	39.5	35.2	38.9
**Religion**											
Hindu	5.6	11.1	23.6	15.3	20.8	18.5	33.8	13.0	38.4	31.0	39.8
Non-Hindu	5.6	0.0	27.8	16.7	22.2	27.8	33.3	16.7	44.4	27.8	33.3
**Caste**											
SC/ST/OBC	5.2	16.9	22.1	11.7	22.1	16.9	36.4	11.7	40.3	22.1	39.0
Others	5.7	7.0	24.8	17.2	20.4	20.4	32.5	14.0	38.2	35.0	39.5
**Family type**											
Nuclear	5.2	9.3	22.2	16.0	20.6	18.6	30.4	13.9	34.0	32.0	36.6
Joint/extended	7.5	15.0	32.5	12.5	22.5	22.5	50.0	10.0	62.5	25.0	52.5
**Employment**											
Not working	4.2	9.5	22.6	12.6	20.5	15.3	31.6	11.6	36.3	28.4	36.8
Working	11.4	13.6	29.5	27.3	22.7	36.4	43.2	20.5	50.0	40.9	50.0
**Marital status**											
Unmarried	14.3	11.4	31.4	25.7	22.9	25.7	34.3	25.7	34.3	40.0	45.7
Married	4.0	10.1	22.6	13.6	20.6	18.1	33.7	11.1	39.7	29.1	38.2

*SC* Scheduled Caste, *ST* Scheduled Tribe, *OBC* Other Backward Classes

### Knowledge and practice of breast examination

**Fig. 4:**
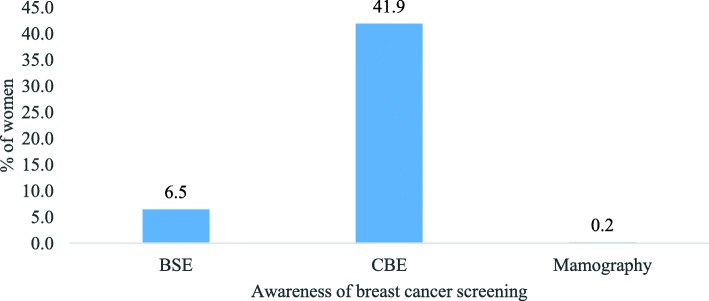
Percentage of women who are aware of breast cancer screening

**Fig. 5:**
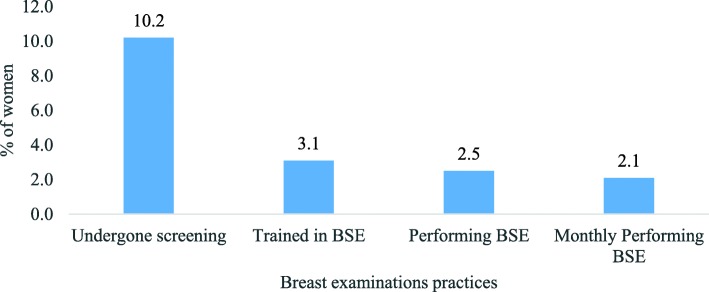
Percentage of women who have undergone screening of breast cancer and performing self-examination of breasts

Of all 480 women, only 6.5% of women knew that breast cancer can be detected through Breast Self-Examination (BSE). Around two out of five (42%) women said cancer in breast can be detected through clinical examination (Fig. 4). Our results showed that around 10% of the women had undergone breast cancer screening. However, only 3.1% were trained in BSE and 2.5% of them were performing BSE. Around 2% of the women were performing BSE monthly (Fig. 5). Almost all women (99.4%) were interested to learn BSE procedure besides three women who were shy of it (not shown in figure).

## Discussion

This study found that breast cancer knowledge among the women in the study area was poor. Only less than half of the women were aware of breast cancer. This proportion was found to be consistent with two of the studies in India conducted in Mumbai (2009) and Delhi (2015) and one studies conducted in Addis Ababa, Ethiopia [15, 19, 25]. On the contrary, a recent study in Mumbai among 18–70 years of women found higher (71%) proportion of knowledge about breast cancer symptoms [26]. Television was found to be the most important source of breast cancer awareness. Our analysis of these 480 women found education as one of the crucial socio-economic factors that influences breast cancer awareness in Mumbai. Our bivariate and multivariate results have also shown consistent results on educational level and breast cancer awareness. A study by Dey et al. (2015) in Delhi also found an association between education and breast cancer awareness [15].

It is important to note that though half of the women were aware of breast cancer, the knowledge of different symptoms was low among these women. Lump in breast is considered as danger sign by most of the women whereas more than half don’t think abnormal discharge/blood, change in shape or size, and change in colour of nipple as danger signs of breast cancer. Another study in Vikroli, Mumbai also found similar results with a very low percentage of women saying the change in shape/ size of breast, discharge from nipple and inverted nipple as danger signs of breast cancer [17]. The study by Somdatta and Baridalyne [16] also found similar outcomes in a resettlement colony of Delhi. In this study, better knowledge of danger signs or symptoms of breast cancer is observed among higher educated and working women than lower educated and not working women respectively. Breast cancer means losing one’s breast(s) was the most common misconception among women.

Like many other Indian studies, this study found the knowledge of risk factors was very low [5, 15–17, 25]. The women in the study identified excessive consumption of tobacco, alcohol consumption and past history as most important risk factors of breast cancer. However, very few women in the community were aware of the risk of breast cancer due to disruption in biological clock like early menarche, late menopause, and hormonal therapy. Further, it is found knowledge of preventable risk factors like hormone replacement therapy, first baby after the age of 30 years, obesity, and use of oral contraceptive pills were low among participants. In this study, we also observed low knowledge of breast screening procedure among women like self-breast examination and mammography. The practice of BSE was very low because they were not trained to about the procedures.

This study is limited to one low socio-economic area of Mumbai, therefore, cannot be generalized to other community. The knowledge of signs, symptoms and risk factors depend on the comprehension capability of the participants during the data collection. Further, the study is cross-sectional in nature and therefore, it is not possible to get any causal relationship between dependent and independent variables.

## Conclusion

This study aimed to assess breast cancer awareness and knowledge of danger signs, symptoms, risk factors and concluded that knowledge of danger signs and risk factors of breast cancer among women in the community was low. Considering the fact that breast cancer has grown as an epidemic in the country, lower knowledge of symptoms and signs may lead to delay in treatment seeking among the women. Although further studies are required at the national level, the lower knowledge of breast cancer among women in one of the advanced metropolises in India calls for greater effort to enhance knowledge of women at the regional and national level. This study calls for intervention to enhance and improve knowledge of breast cancer among women in the particular region with a special reference to women with low educational level and marginalised community. Effective media platform like television can be used to promote breast cancer awareness and breast self-examination practices. Advocacy and health education related to breast cancer awareness and screening methods and their accessibility needs to be strengthened in government programme with focus in lower socio-economic areas. Further, preparing appropriate and specific content for health education with an emphasis on preventable risk factors and lifestyle modification will enhance the awareness level and strengthen practices for prevention and early detection breast cancer.

## Supplementary information

**Additional file 1.**

## Data Availability

The raw data used in this research is available with the researchers. Please send your inquiries to the corresponding author.

## Abbreviations

BC: Breast Cancer
GLOBOCAN: Global Cancer Incidence, Mortality and Prevalence
BSE: Breast Self-Examination
CBE: Clinical Breast Examination
SC: Scheduled Caste
ST: Scheduled Tribe
OBC: Other Backward Classes
AOR: Adjusted Odds Ratio
CI: Confidence Interval
UK: United Kingdom
OR: Odds Ratio
BMI: Body Mass Index
MCGM: Municipal Corporation of Greater Mumbai
MHADA: Maharashtra Housing and Area Development Authority
ICMR: Indian Council of Medical Research
NIRRH: National Institute for Research in Reproductive Health
