# Supplementary material for: Knowledge of symptoms and risk factors of breast cancer among women: a community based study in a low socio-economic area of Mumbai, India
Source: BMC Womens Health. 2020 May 18;20:106. doi: 10.1186/s12905-020-00967-x (PMC7236367; doi:10.1186/s12905-020-00967-x)
Supplement: Supplementary file 1 — Additional file 1. [file 12905_2020_967_MOESM1_ESM.docx]

**National Institute for Research in Reproductive Health (ICMR-NIRRH), Parel, Mumbai**

Confidential for research purpose

**Intervention to Increase Breast Cancer Awareness and Breast Self-Examination among Women in the Community- A Multiple Approach**

|  |  |  |
| --- | --- | --- |

**Interview Schedule for Women (Baseline Survey)**  Sr. No.

| **Sr. No.** | **Questions** | **Coding Categories** |
| --- | --- | --- |
| 1. | How old are you? (Age in completed years) |  |
| 2. | Have you ever attended school? | Yes ……………1  No …………….2 **🡪 Skip to 4** |
| 3. | What is the highest standard you completed?  “NUMBER OF YEARS OF SCHOOLING” | ________________ |
| 4. | What is your religion? | Hindu ……………………1  Muslim .…..…………..…..2  Christian…………………..3  Sikh………………….……4  Buddhist/Neo Buddhist…………….…….5  Jain…………………….….6  Parsi/Zoroastrian………….7  No Religion……………….9  Others______________ 98 |
| 5. | Do you belong to a scheduled caste, scheduled tribe, other backward class or none of these? | Scheduled Caste ………...1  Scheduled Tribe…………..2  OBC ……………….….….3  None of them……………..4 |
| 6. | Type of family | Nuclear family …………...1  Joint family...……………..2  Extended family ……….....3 |
| 7. | Are you currently employed? | Yes ………………………1  No………………………2**🡪 Skip to 8** |
| 7a. | If employed, what is your occupation? | Not working/House wife …0  House maid servant……….1  Clerk………………………2  IT Industry………………...3  Nurse……………………...4  Teacher……………………5  Police ……………………..6  Other (specify) ……………7 |
| 8. | Bathroom facility (Type) | Mori (Local)………………1  Separate bathroom………...2  Common bathroom………..3  Other………………………4 |

| **Sr. No.** | **Questions** | **Coding Categories** |
| --- | --- | --- |
| 9. | Are you currently married? | Yes ……………1  No …………….2 **🡪 Skip to 19** |
| 10. | How old were you when you got married? |  |
| 11. | Have you ever given birth? | Yes ……………1  No …………….2 **🡪 Skip to 15** |
| 12. | How old were you at the time of first childbirth? |  |
| 13. | How many sons and daughters are alive? | Sons _________  Daughters _________ |
| 14 | Duration of breastfeeding for each child (in months)  (WRITE ‘00’ FOR NO BREASTFEEDING) | Child 1…………….  Child 2…………….  Child 3…………….  Child4…………….. |
| 14a. | Have you taken any galactogogues (*shatavari* to increase breast milk)? | Yes ………………………1  No …… …………………2 |
| 15. | Have you ever had any medical abortion? | Yes ………………………1  No ………………………2 **🡪 Skip to 16** |
| 15a. | If yes, number of medical abortions?  “MENTION NUMBER OF ABORTION/S” | Numbers___________ |
| 16. | Have you ever used oral pills to delay pregnancy? | Yes ………………………1  No ………………………2 **🡪 Skip to 17** |
| 16a. | If yes, duration of use  “MENTION NUMBER OF MONTHS” | Months…………… |
| 17. | Are you currently using oral pills to delay pregnancy? | Yes ………………………1  No ………………………2 **🡪 Skip to 18** |
| 17a. | If yes, duration of use  “MENTION NUMBER OF MONTHS” | Months…………… |
| 18. | Have you ever used emergency contraceptive pills to avoid pregnancy? | Yes ………………………1  No …… …………………2 |
| 18a. | If yes, How many times  “MENTION NUMBER OF TIMES” |  |
| 19. | Do you have regular menstrual cycle?  If yes, Mention last menstrual period (LMP) date  (USE DD/MM/YYYY FORMAT) | Yes ………………………1  No …… …………………2  **----------------------------------** |

| **Sr. No.** | **Questions** | **Coding Categories** |
| --- | --- | --- |
| 20. | Do you wear underwire bra? | Yes ………………………1  No …… …………………2 |
| 20a. | Remove bra during night (sleeping) | Yes ………………………1  No …… …………………2 |
| 21. | Where do you go for your general health check-up? | Public clinic………...…....1  Private clinic….……...…..2  Poly clinic………...……...3  Ayurveda doctor……........4  Homeopath doctor…….…5  Home remedies………..…6  Others ……………………7 |
| 21a. | How far is the maternity home from your place?  “MENTION NUMBER OF KILOMETERS” | __________________Km. |

| **Knowledge of breast cancer symptoms** | | | |
| --- | --- | --- | --- |
| 22. | Have you heard of breast cancer? | Yes ……………1  No ……………2**🡪Skip to 42** |  |
| 23. | From where did you get information about breast cancer?  “RECORD ALL” | Friend…………………….A  Neighbour…………….….B  Relative…………………..C  Doctor…………………....D  Radio………….……….…E  Television………..….…...F  Newspaper/Magazine…....G  Poster………….…………H  Other (specify)……….…..I |  |
|  | ***Now I am going to ask you about the signs & symptoms of Breast Cancer*** | |  |
| 24. | Change in the shape / size of nipple could be a sign of breast cancer | Yes ………………………1  No …………………….…2 |  |
| 25. | Pain in one of breasts could be a sign of breast cancer | Yes ………………………1  No …………………….…2 |  |
| 26. | Abnormal discharge/blood from nipple could be a sign of breast cancer | Yes ………………………1  No …………………….…2 |  |

| **Sr. No.** | **Questions** | **Coding Categories** |
| --- | --- | --- |
| 27. | A lump in breast could be a sign of breast cancer | Yes ………………………1  No …………………….…2 |
| 28. | Change in skin color, could be a sign of breast cancer | Yes ………………………1  No …………………….…2 |
| 29. | A lump under armpit could be a sign of breast cancer | Yes ………………………1  No …………………….…2 |
| 30. | Changes in the shape / size of breast could be signs of breast cancer | Yes ………………………1  No …………………….…2 |
| 31. | Breast cancer can be hereditary | Yes ………………………1  No …………………….…2 |
| 32. | Breast cancer can be present in absence of pain | Yes ………………………1  No …………………….…2 |
| 33. | Breast cancer is curable if detected in early stages | Yes ………………………1  No …………………….…2 |
| 34. | Woman with big breast get breast cancer | Yes ………………………1  No …………………….…2 |
| 35. | Use of antiperspirants or deodorants causes breast cancer | Yes ………………………1  No …………………….…2 |
| 36. | Trauma to breasts cause breast cancer | Yes ………………………1  No …………………….…2 |
| 37. | Breast cancer is communicable | Yes ………………………1  No …………………….…2 |
| 38. | Breast cancer means losing one’s breast(s) | Yes ………………………1  No …………………….…2 |

| **Sr. No.** | **Questions** | **Coding Categories** |
| --- | --- | --- |
| 39. | What are the risk factors of breast cancer?   1. Menstruation at an early age (<12 yrs) 2. Woman having late age at menopause 3. Excess use to tobacco 4. Excess use to alcohol 5. Woman who had multiple induced abortion/s 6. Nulliparity 7. Woman having first baby after age 30 years 8. Woman used oral contraceptives/pills 9. Woman who is obese 10. Woman consumes high fat 11. Woman undergone hormone replacement therapy 12. Woman who has not given breastfeeding to the child 13. Woman with family history of breast cancer 14. Woman having past history of breast cancer | 1. Yes…….1 No………2 2. Yes…….1 No………2 3. Yes…….1 No………2 4. Yes…….1 No………2 5. Yes…….1 No………2 6. Yes…….1 No………2 7. Yes…….1 No………2 8. Yes…….1 No………2 9. Yes…….1 No………2 10. Yes…….1 No………2 11. Yes…….1 No………2 12. Yes…….1 No………2 13. Yes…….1 No………2 14. Yes…….1 No………2 |
| 40. | What are different methods of breast cancer detection?  “RECORD ALL” | Breast self-examination…..A  Clinical examination…..….B  Mammography…………....C  other (specify) __________D |
| 41. | Have you ever undergone breast cancer screening? | Yes ………………………1  No …… …………………2 **🡪Skip to 42** |
| 41a. | If yes, where? (Specify) ________________________________________ | |
| 41b. | If yes to Q 41, Status of screening | Normal…………..……….1  Other (specify)**----------------** |
| 42. | Would you like to undergo clinical examination for breast cancer? | Yes ……………1**🡪Skip to 42b**  No …… ………2 |
| 42a. | if no reasons______________________________ |  |
| 42b. | If yes, which day and time would you prefer for examination ______________________________ | Day **-----------------------------**  Time __________________ |
| 43. | Have you ever been trained for breast self-examination (BSE)? | Yes ……………1  No……………2**🡪Skip to 44** |
| 43a. | If yes, who trained you on BSE? (Specify)__________________________ | |
| 43b. | Do you practice breast self-examination? | Yes ………………………1  No …… …………………2 |
| 43c. | If yes, how often (Frequency)? | Monthly …………………1  Once in 3 months ……..…2  Once in 6 months………...3  Yearly………………..…..4 |
| 43d. | If no, Reason_______________________________ | |

| **Sr. No.** | **Questions** | **Coding Categories** |
| --- | --- | --- |
| 44. | Would you like to get breast self-examination training? | Yes ……………1**🡪Skip to 45**  No………………2 |
| 44a. | If no, why? Specify ____________________________________________ | |
| 45. | Would you like to have more information about breast cancer? | Yes ………………………1  No …… …………………2 |
| 46. | Which method of communication would you prefer for breast cancer education?   1. Gather people for health education 2. Television 3. Breast cancer survivors teach about breast cancer 4. Pamphlets 5. Street plays 6. Video or film 7. Health care provider provides information on breast cancer | 1. ….. 2. ….. 3. ….. 4. ….. 5. ….. 6. …… 7. …… |

Please inform her details (day, time and venue) about group session on breast self-examination and clinical breast examination.

Date:____________________ ________________________

Name of Investigator & Signature
